# Factors influencing the utilization of Focused antenatal care services during pregnancy, a study among postnatal women in a tertiary healthcare facility, Ghana

**DOI:** 10.1002/nop2.569

**Published:** 2020-07-26

**Authors:** Kennedy Diema Konlan, Joel Afram Saah, Roberta Mensima Amoah, Abdul Razak Doat, Iddrisu Mohammed, Juliana Asibi Abdulai, Kennedy Dodam Konlan

**Affiliations:** ^1^ Department of Public Health Nursing School of Nursing and Midwifery University of Health and Allied Sciences Ho Ghana; ^2^ Department of Public Health School of Allied Sciences University for Development Studies Tamale Ghana; ^3^ Nursing and Midwifery Training College Tamale Ghana; ^4^ Surgical Department, Urology Tamale Teaching Hospital Tamale Ghana; ^5^ Department of Nursing West End University College Accra Ghana

**Keywords:** antenatal, factors, focused antenatal care, influencing, maternal health, pregnancy, utilization

## Abstract

**Aim:**

To assess the factors that influence the utilization of FANC services among pregnant women.

**Methodology:**

A cross‐sectional quantitative study conducted among 210 postnatal women in Ho Teaching Hospital. Data were entered into Microsoft excel, cleaned and transported to SPSS and analysed. Cross tabulations were used to explore associations between variables.

**Results:**

The respondents indicated that FANC would enable them to receive comprehensive ANC (74.8%). Higher parity was significantly associated with low utilization of FANC (*p* = .028). Long distance to the health facility, seeking permission to use FANC was significantly associated with low utilization of FANC (*p* < .001). Fear associated with witchcraft was associated with low FANC utilization (*p* < .001).

## 1 INTRODUCTION

Most maternal deaths (66%) occurred in sub‐Saharan Africa, while 99% of the maternal deaths occurred in low‐ and middle‐income countries as most could have been prevented (WHO, 2014). The primary causes of these maternal deaths were haemorrhage, hypertensive diseases of pregnancy and sepsis and indirect causes, mostly due to interaction between pre‐existing medical conditions and pregnancy (WHO, 2014). In 2001, the World Health Organization (WHO) issued guidance on a new model of Antenatal Care (ANC) called goal‐oriented or focused antenatal care (FANC), for implementation in developing countries (Villar et al., 2001) including Ghana. Focused ANC means that pregnant women attend a minimum of four scheduled ANC visits and receive all the WHO recommended comprehensive packages by skilled healthcare providers (WHO, 2014). This new model hinges on the quality of services than quantity of services received by expectant mothers. Focused antenatal care eliminated the traditional risk assessments and instead stressed on helping women to maintain normal pregnancies (WHO, 2010). In response to this evidence, several countries in sub‐Saharan Africa moved to adapt FANC as a way of promoting the health and survival of mothers and babies. Globally, there is a remarkable decline in maternal mortality ratio—MMR (WHO, 2012). Despite this recent decline, Sub‐Saharan Africa has the highest MMR in the world, even though there are strategies and interventions that prioritize maternal health (Hogan et al., 2010; WHO, 2012).

In sub‐Saharan Africa, maternal mortality ratio (MMR) was estimated to be 500 per 100,000 live births in 2010. To kerb this high rate of maternal deaths, it is estimated that an annual decline in maternal mortality of 5.5% is needed; however, between 1990–2010 the annual decline was only 1.7% in the sub‐Saharan region (WHO, 2012). The use of antenatal services in some parts of Africa and the developing world has not been encouraging. Usually, pregnant women come late for the services and make less than the recommended number of focused antenatal care (FANC) visits (Magadi, Madise, & Rodrigues, 1999; Ndidi & Oseremen, 2010). The maternal mortality ratio in Ghana currently stands at 308 deaths per 100,000 live births (WHO, 2019), though this figure is relatively lower compared to the number of maternal deaths that occurred over a decade ago (WHO, 2019).

The role of ANC services in reducing maternal mortality in Ghana has been reported by the World Health Organization (WHO, 2019), yet some women do not attend ANC during pregnancy. The rate of ANC visits varies among pregnant women; this is evident in a study conducted in the Bunkpurugu district of Ghana, where 1.6% of pregnant women attended ANC once, 12.9% between two to four times, 22.6% attended at least five times and the remainder attended more than five times (Konlan et al., 2016). In a few African countries like Nigeria, 77% of the pregnant women start utilizing focused antenatal care in the second trimester (Ndidi & Oseremen, 2010), while in Kenya 45% in the third trimester (Magadi et al., 1999). In Malawi, 48% of the pregnant women start utilizing focused antenatal care in the second trimester (Malawi Demographic and Health Survey, 2010). In terms of number of visits, in the developed countries, 97% of pregnant women make at least one ANC visit and 99% of these pregnant women deliver with skilled birth attendants (Mrisho et al., 2009). The contrary, however, is that, in developing countries, including Ghana, 49% of pregnant women make at least one FANC visit and often two‐thirds of these women deliver with unskilled birth attendants (Raatikainen, Heiskanen, & Heinonen, 2007).

In incorporating FANC into maternal and child health services in Ghana, the reproductive health policy and antenatal clinic (ANC) guidelines were revised to include early detection and treatment of all complications arising during pregnancy. Emphasis was laid on assessing birth preparedness and complication readiness, prevention of malaria in pregnancy and prevention of mother to child transmission of HIV (PMTCT). The ANC schedule was reduced from thirteen (13) visits to four (4) comprehensive, personalized visits. FANC concentrates on the quality of care rather than the number of visits and ensures good health during labour and delivery. This puts the relationship of the care provider and the client central to success. The kind of relationship established by the midwife and client such as kindness, respect, privacy, confidence level during antenatal is central to the amount of trust established and the extent of care activities implemented in the absence of the midwife, especially at home (Konlan, Kombat, Japiong, & Konlan, 2018). Developing countries are struggling to achieve quality FANC provision, particularly in rural and peri‐urban areas. Competition for staff and money as well as poor communication with other programmes or components (malaria, HIV, emergency obstetric care) can be found at different levels of the health system, particularly where policies are ill‐defined (Baffour‐Awuah, Mwini‐Nyaledzigbor, & Richter, 2015). Poor communication among healthcare providers, as well as the perception of some women, families and communities about pregnancy being a natural process of life, may lead to underestimation of the importance of FANC (Baffour et al., 2015).

In addition, lack of knowledge about the extent and impact of traditional household and community beliefs and customs, suboptimal maternal nutrition and infant feeding practices as well as the attitudes and behaviours of healthcare providers in FANC clinics such as failing to respect the privacy, confidentiality and traditional beliefs of the women negatively influence the use of FANC (Baffour et al., 2015). The belief and role of witches, a supernatural force that has the tendency to control and manipulate individuals and family towards an evil gain, is a widespread belief in most parts of the African societies. The general belief in this particular evil spirit has the potential to prevent early disclosure of pregnancy and may limit the chance of seeking early focused antenatal care service. Ethnographic studies from Mozambique and southern Tanzania illustrated, for example, that women at an early stage of pregnancy delayed ANC initiation purposely to protect the unborn from witchcraft and sorcery attacks of jealous neighbours and kin (Chapman, 2003; Gross, Alba, Glass, Schellenberg, & Obrist, 2012; Haws et al., 2010). While these factors have been well documented under some jurisdictions, imperative research in the Volta region of Ghana has not specifically documented those factors that influence the utilization of FANC services among pregnant women seeking services in the Ho Teaching Hospital.

### 1.1 Aim

This study assessed the factors that influence the use of focused antenatal care services during pregnancy among postnatal women in the Ho Teaching Hospital of the Volta region of Ghana.

## 2 METHODOLOGY

### 2.1 Study design

A cross‐sectional descriptive study that assessed the views of postnatal women on the factors that influenced the utilization of FANC services during pregnancy.

### 2.2 Setting and population

This study was conducted in the Ho teaching hospital. In the year 2018, the hospital was upgraded from the level of a regional hospital to a teaching hospital following the establishment of the school of medicine at the University of Health and Allied Sciences. The study population for this study were postnatal women who had received ANC services at the Ho teaching hospital. The Ho teaching hospital is located along the Ho to Aflao road. The approximately 400‐bed capacity facility has nine wards. The facility also has three major theatres, a mortuary, laundry, and sterilizing department. The hospital serves as the main referral point for smaller facilities in the region and offers other services such as reproductive and child health, family planning services, dental, ear, nose and throat, eye, nutrition rehabilitation, antiretroviral unit, planned home birth, diabetic clinic and a dialysis unit. The facility also has a dispensary, laboratory, catering, public health and general administrative units, as well as an outpatient department (OPD). There are approximately 45 midwives in the Obstetrics & Gynaecology (O&G) department, two O&G specialists and two medical doctors with four house officers who are rotated every 4–5 months. The midwives and doctors run a three‐shift duty within 24 hr with a specialist on call at every point in time. Postnatal women who sort services in the Ho teaching hospital were recruited into the study as they came for routine postnatal service for their babies.

### 2.3 Sampling and sample size

The sample size (*n*) of the study was determined by the Snedecor and Cochran (1989) sample size formula: $n=\frac{Z^{2}pq}{d^{2}}$ where *Z* = standard normal score corresponding to 95% confidence level = 1.96, *p* = proportion of women in fertility age (0.154), *q* = 1 − *p d* = degree of precision (margin of error), *N* = desired sample size $n=\frac{1.92^{2}*0.154\left(1‐0.154\right)}{0.05^{2}}$ sample size (*N*) = 200.2 ~ 200 Non‐response rate = 5% of *n* ⸫ *N* = 210.

The sampling for this study was systematic sampling method. The estimated sample fraction was calculated to be 3.5. Research assistants recruited every third person, and in instances the person did not consent, the fourth person was recruited. The postnatal service refers to the care rendered to the baby and mother within the period from conception to 6 weeks postconception. All postnatal women who met these inclusion criteria were included in the study.

### 2.4 Tool and data collection

A questionnaire was used for data collection. The questionnaire contained 38 items, mainly on demographic, utilization and factors that influence the utilization of ANC services during pregnancy. To ensure that the research questions were not ambiguous, the questionnaire was pre‐tested on forty postnatal women in the Ketu South municipal hospital, Aflao. The data collected were subjected to a reliability test on SPSS version 22. This was done to ascertain the respondents' general reaction and, particularly, interest in answering the questionnaire. The questionnaire was modified until it produced a *Cronbach's alpha coefficient* of 0.801. It can therefore be concluded that the instrument had a high reliability in measuring needed data for the study. Responses from the people showed that the questionnaire was clear and could be understood by others. The questionnaire was made of open‐ended questions and closed‐ended questions. Some closed‐ended questions were largely dichotomous, while others had multiple choices.

Research assistants received 2 days of training on research ethics and on the study tool and data entry. The four research assistants collected data between the hours of 8 a.m. and 4 p.m. each working day during the 3‐week period of data collection. The data collected daily were entered into Microsoft excel.

On each day, trained research assistants visited the postnatal clinic of the Ho Teaching Hospital and recruited participants in the study. The data were collected within 3 weeks. Women who sort postnatal services for their babies at the postnatal clinic were recruited to respond to a pre‐tested questionnaire after they had received service. It took an average of 15 min to complete a questionnaire. In instances the respondent could not read and write, the research assistant aided them to complete the questionnaire.

### 2.5 Data analysis

The data were checked for completeness and appropriateness of responses. The data were then entered daily into Microsoft Excel 2016 version and cleaned and transported to statistical package for social sciences version 22 for analysis. Data were analysed as descriptive statistics, and some cross tabulation was done between some demographic variables and the factors that influence the utilization of FANC services among pregnant women. Socio‐cultural factors were also cross tabulated against the number of ANC visits made by pregnant women; *p* < .05 was considered statistically significant.

The number of FANC visits was categorized into dichotomous variables; FANC visits less than four attendance (<4) denoting low utilization and FANC visits of four or more attendance (≥4) denoting adequate utilization. Identification of demographic and socio‐cultural variables associated with low utilization was carried out using cross tabulations. Statistical significance, evaluated at 0.05 levels, was assessed with SSPS. Explanatory variables were dichotomized prior to running cross tabulations; Yes or No (0 or 1) responses were assigned to some socio‐cultural variables. Demographic variables such as marital status (married and unmarried), parity (nulliparous and multiparous), ethnicity, religion (Christianity and other), gravidity (primigravidae and muiltigravidae) and occupation were also dichotomized. Participating mothers’ responses to open‐ended questions about barriers associated with low utilization were put into themes and thereafter responses were coded and dichotomized (Yes or No).

### 2.6 Ethical considerations

Research ethics were obtained from the institute for health research from the University of Health and Allied sciences (UHAS‐REC A.1 (19) 17–18). The research was scientifically reviewed by the scientific review committee of the School of Nursing and Midwifery of the University of Health and Allied Sciences, on whose recommendation research ethics certificate was issued. Permission was sort from the management of the Ho Teaching Hospital prior to the commencement of data collection. Research respondents each gave written and verbal consent to participate in the study. The template of the Institute of Health Research from the University of Health and Allied Sciences in Ho's template on research ethics, consent form was used as a guide in developing the consent form. Participants who could not sign were made to thumbprint on the section for signature.

## 3 RESULTS

The age range of postnatal women showed 20.5% within 16–20 years, 29.5% 21–25 years, 26.2% 26–30 years, 17.1% 31–35 years and 6.7% 36–40 years. Most women were married (47.6%), as 19.5% were cohabiting. A considerable proportion were Ewes (58.6%), Akans (18.6%) and Ga‐Adangbes (11.4%), while Christians were 73.8%. Few women (17.1%) had no formal education. Also, 30.0% were primipara, 33.8% birthed twice and 18.6% birthed thrice. Trading (46.2%) was the major income‐generating activity among the postnatal women.

Most postnatal women (47.1%) have no awareness of FANC. The major sources of information on FANC were from midwives (51.4%), relatives (18.9%), radio (18.0%), traditional birth attendants (0%) and other sources like friends were 11.7%. The responses for reasons behind FANC clinic visit included when there is a problem in the pregnancy (20.5%), as 52.9% indicated when there is no problem. An appreciable proportion (61.3%) of postnatal women indicated that FANC is useful in establishing a rapport between the pregnant mother and the midwife. Respondents (62.2%) agreed with the notion that antenatal care would help in early detection of risks associated with pregnancy. Also, 64.9% showed that FANC would assist the health worker to distribute information, education and communication materials. The findings showed that 7.2% disagreed and 74.8% of the respondents also agreed with the fact that the FANC would enable them to receive tetanus toxoid vaccine, vitamin A, iron supplementation, insecticide‐treated nets, intermittent preventive treatment and hookworm treatment (Table 1).

**TABLE 1:** Demographic characteristics (*N* = 210)

Variables	Characteristics	*f*	%.
Age	16–20	43	20.5
	21–25	62	29.5
	26–30	55	26.2
	31–35	36	17.1
	36–40	14	6.7
Marital Status	Married	100	47.6
	Single	49	23.3
	Divorced	16	7.6
	Widowed	4	1.9
	Cohabiting	41	19.5
Ethnic Group	Ewe	123	58.6
	Akan	39	18.6
	Ga‐Adangbe	24	11.4
	Other	24	11.4
Religion	Christian	155	73.8
	Islam	27	12.9
	Traditional African	19	9.1
	Other	9	4.3
Educational Level	Primary	56	26.7
	Junior high school	56	26.7
	Senior high school	36	17.1
	Tertiary	26	12.4
	None	36	17.1
Occupation	Business	38	18.1
	Trader	97	46.2
	Farmer	12	5.7
	Student	11	5.2
	Civil servant	22	10.5
	Other	30	14.3
Partner Occupation	Business	29	20.6
	Civil servant	50	35.5
	Farmer	27	19.2
	Student	8	5.7
Parity	One	63	30
	Two	71	33.8
	Three	39	18.6
	Four	23	11.0
	More than four	14	6.7

Most of the postnatal mothers (72.9%) attended FANC at some point during their previous pregnancies. Postnatal women (27.6%) initiated FANC visits between 0–3 months, 27.1% 4–6 months and 12.9% 7–9 months, while 32.4% had no idea when they started. The number of visits throughout the pregnancy was varied as 51.0% made fewer than four visits, 7.6% made four visits, 13.3% made more than four visits and 28.1% made one visit. Age was marginally (*p* = .028) related to the number of visits, with those between 16–20 years, 31–35 years as well while 36–40 years used FANC more. A greater percentage attended at least four times compared with 21–25 and 26–30 who used FANC less as the greater percentage attended less than four times (Table 2).

**TABLE 2:** Knowledge on FANC (*N* = 210)

Responses	*f*	%
Definition Of FANC		
Evidence‐based and goal directed, family‐centred and quality rather than quantity of visits	66	31.4
Individualized care with high quantity of care	45	21.4
No idea	99	47.2
Sources Of Information		
Midwife	57	51.4
Radio	20	18
Traditional birth attendants	0	0
Relatives	21	18.9
Others	13	11.7
Reason For Visit		
When there is a problem	43	20.5
When there is no problem	111	52.9
No response	56	26.6
Benefits Of FANC		
Establishing rapport between pregnant mother and antenatal care provider	68	61.3
For early detection of risks associated with pregnancy	69	62.2
Assist the provider to give individualized health education on the importance of FANC	72	64.9
To receive preventive interventions such as tetanus immunizations, iron and folic acid supplementation, malaria treatment (SP), insecticide‐treated bed nets.	83	74.8

Married postnatal women and those cohabiting used FANC more compared with the single, divorced and widowed (*p* = .460). Likewise, Ewes used FANC more compared with the Akans and Ga‐Adangbes (*p* = .357). Christians and Moslems used FANC more than the Traditional African (*p* = .327). Respondents with secondary, tertiary or no educational background used FANC more compared with those with primary and junior high educational background (*p* = .003). On the part of the participants, occupation, traders, students and civil servants patronized FANC more compared with those involved in business, farming and other occupations (*p* = .003). On the other hand, respondents whose partners were businessmen, civil servants and students patronize FANC more compared with those whose partners were farmers and involved or other occupations (*p* = .149). Parity was significantly associated with the number of visits to the FANC (*p* = .003). Postnatal women with a single child alive utilized FANC compared to other women (*p* = .000).

Participating women who reported having problems of money for transportation made less than required (≥4) visits to FANC (*p* = .000). Long distance also significantly influenced the number of visits to FANC (*p* = .000). Participating women who reported having problems of desirability made less than required (≥4) visits to the FANC (*p* = .000). Waiting to obtain permission was also a factor which significantly resulted in fewer visits to FANC (*p* = .000). Participating women who reported having concerns that there will be no midwife available made less than required (≥4) visits to FANC (*p* = .000). Participating women who reported having problems with limited transportation options made less than required (≥4) visits to FANC (*p* = .000) (Table 3).

**TABLE 3:** Association of demographic factors and the utilization of FANC by postnatal women (*N* = 210)

Responses	Had less than 4 ANC attendance (<4)	Had 4 or more ANC attencace (≥4)	*p* value
Age			
16–20	32 (19.28)	11 (25.0)	.028
21–25	55 (33.1)	7 (15.9)	
26–30	44 (26.5	11 (25.0)	
31–35	28 (16.9)	8 (18.2)	
36–40	7 (4.2)	7 (15.9)	
Marital status			
Married	76 (45.8)	24 (54.5)	.460
Single	40 (24.1)	9 (20.5)	
Divorced	15 (9.0)	1 (2.3)	
Widowed	4 (2.4)	0 (0.0)	
Cohabiting	31 (18.7)	10 (22.7)	
Ethnic distribution			
Ewe	94 (56.6)	29 (65.9)	.357
Akan	32 (19.3)	7 (15.9)	
Ga‐Adangbe	22 (13.3)	2 (4.5)	
Other	18 (10.8)	6 (13.6)	
Religion			
Christian	118 (71.1)	37 (84.1)	.327
Traditional African	23 (13.9)	4 (9.1)	
Islam	16 (6.6)	3 (6.8)	
Other	9 (5.4)	0 (0.0)	
Education			
Primary	50 (30.1)	6 (13.6)	.003
Junior High	50 (30.1)	6 (13.6)	
Secondary	23 (13.9)	13 (29.6) 8 (18.2)	
Tertiary	18 (10.8) 25 (15.1)	11 (25.0)	
None			
Occupation			
Business	33 (19.8)	5 (11.4)	.003
Trader	72 (43.4)	25 (56.8)	
Farming	12 (7.2)	0 (0.0)	
Student	5 (3.0)	6 (13.6)	
Civil Servant	16 (9.6)	6 (13.6)	
Others	28 (16.8)	2 (4.6)	
Partners’ occupation			
Business	21 (19.6)	8 (23.5)	.149
Civil Servant	34 (31.8)	16 (47.1)	
Farming	23 (21.5)	4 (11.7)	
Student	5 (4.8)	3 (8.8)	
Other	24 (22.4)	3 (8.8)	
Parity			
One	43 (25.9)	20 (45.5)	.003
Two	66 (39.8)	5 (11.4)	
Three	30 (18.1)	9 (20.5)	
Four	17 (10.2)	6 (13.6)	
More than four	10 (6.0)	4 (9.1)	
Number of children alive			
One	51 (30.7)	23 (52.3)	.000
Two	65 (39.1)	3 (6.8)	
Three	25 (15.1)	9 (20.5)	
Four	16 (9.6)	5 (11.4)	
More than four	9 (5.4)	4 (9.1)	

Fear that wizards may terminate the pregnancy was significantly associated with the number of visits women made (*p* = .000). In addition, long waiting hours at health facilities was a factor which statistically significantly resulted in greater number making the least number of visits to FANC (*p* = .000). Similarly, lack of knowledge and no time to attend FANC was a factor which statistically significantly resulted in greater number making the least number of visits to FANC (*p* = .000). Respondents who reported having problems of being shy and receiving poor attitudes from staff made less than required (≥4) visits to the FANC (*p* = .000). Those who reported personal reason (just not wanting to make lots of visits) were also significantly less likely to attend FANC (*p* = .000). Besides, poor amenities were a component that resulted in a greater number of respondents making the required number of visits to FANC (*p* = .000) (Table 4).

**TABLE 4:** Socio‐cultural factors associated with utilization of FANC (*N* = 210)

Variable	Number of ANC attendance		*p* value
	Had less than 4 ANC attendance (<4)	Had 4 or more ANC attendance (≥4)	
Transport money	1 (0.6)	0 (0.0)	.000
Long distance	8 (4.8)	1 (2.3)	.000
Desirability	1 (0.6)	0 (0.0)	.000
Need permission to start FANC	24 (54.6)	11 (6.6)	.000
Concern that there may not be a midwife	1 (2.3)	0 (0.0)	.000
Limited transportation options	5 (3.0)	0 (0.0)	.000
Fear that witches may terminate the pregnancy	9 (5.4)	0 (0.0)	.000
Long waiting hours	6 (3.6)	0 (0.0)	.000
No knowledge	14 (8.4)	0 (0.0)	.000
No time	6 (3.6)	0 (0.0)	.000
Poor amenities at the facility	0 (0.0)	2 (4.6)	.000
Poor staff attitude	7 (4.2)	4 (9.1)	.000
Shyness	7 (4.2)	0 (0.0)	.000
No barriers	91 (54.8)	12 (27.3)	.000

## 4 DISCUSSION

This study assessed the factors that influence the utilization of FANC services in the Ho Teaching Hospital located in the Volta Region of Ghana. Age in general influenced utilization of FANC among postnatal mothers; the age group of 21–25 made the fewest FANC visits (33.1%). The influence of age on the utilization of antenatal care services among pregnant women has been largely reported by many researchers all over the world. For example, Weller, Eberstein, and Bailey (1987) indicated that age alone may not influence uptake of antenatal care services, but pregnancy desirability as mistimed or unwanted pregnancies are associated with irregular and late FANC utilization. Also, Magadi, Madise, and Rodrigues (2000) reported that younger women were more likely to delay starting prenatal care and made fewer antenatal visits. In Bangladesh, Bhatia and Cleland (1995) as cited by Nyarko et al. (2006) show that age is associated with low utilization of antenatal care, particularly among women older than 18 years. In Ghana, younger mothers have the pregnancies unplanned for and sometimes may not be aware of the pregnancy. The women in this age bracket, however, have higher risk pregnancy complications. This finding reinforces the need to intensify advocacy messages aimed at promoting FANC utilization among women of reproductive age group.

The number of visits throughout the pregnancy was varied as 51.0% made less than four visits, 7.6% made four visits, 13.3% made more than four visits and 28.1% had only one visit. WHO recommended that a woman without complications should have at least four antenatal care visits, the first of that should take place during the first trimester (WHO, 2014). Focused antenatal care (FANC) is personalized care provided to a pregnant woman, which emphasizes on the women's overall health status, her preparation for childbirth and readiness for complications or it is timely, friendly, simple safe services to pregnant women (Mamba, Muula, & Stones, 2017).

There was a negative relationship between parity and utilization of FANC (0.003), with multiparous women making fewer visits to FANC than nulliparous women. The state of fear and general feeling of insecurity among primiparous women might account for this disparity in seeking antenatal care. Also, recent public health interventions and education has concentrated mainly on women who seek antenatal care. With information, such cadre has a higher chance of implementing learnt information than a primiparous woman. However, in Ghana when pregnancies were cared for based on risk assessment, primiparous women were classified as high‐risk pregnancies. In Kenya, Magadi et al. (2000) demonstrated that higher parity was associated with low utilization of FANC services. Again, Ethiopian multiparous mothers were more likely to use FANC services than nulliparous counterparts (Mekonnen & Mekonnen, 2003). These findings allude to the fact that there is still the need to continue with community sensitization on the need to maximize FANC regardless of parity. There was no significant relationship between educational level of women and the utilization of FANC services. Ho has largely been an urban community with many social interventions that target all cadres of women, especially during pregnancy. During antenatal care, local dialects are used to provide health education to ensure total participation of all women. Besides, local news outlets provide pregnancy education in local dialects on news airwaves. Matsumula and Gubhaju (2001) indicated that low utilization of FANC is associated with low education. The lack of effect of education on utilization of FANC in this study may be due to high levels of low education among the participating women making it hard to show a difference. Moreover, Pallikadavath, Foss, and Stones (2004) argue that education assists in the adequate utilization of FANC service.

There was no significant relationship between marital status and the utilization of FANC services among postnatal women. This may be an indication that the continued empowerment of women, especially in peri‐urban areas, is yielding some dividends as women without husbands or partners equally take responsibility for the health of themselves and babies. This finding differs with Tann et al. (2007) that unmarried status influenced less uptake of antenatal care services. The findings of the study elucidated that occupational status of both the husband and the participating mothers was not associated with low utilization of FANC services.

Almost all the participating mothers indicated that socio‐cultural factors played a greater role in utilization of FANC services. Distance to the health facility statistically significantly determined both the probability and frequency of attending FANC clinics (*p* = .000). Long distance to the health facility is indicated as highly associated with few visits. Some women have to travel over long distances to seek health care and this coupled with deplorable roads and inefficient transportation system will invariably influence women's likelihood of attending antenatal services. An interesting finding was the prevalence of superstition related to pregnancy (5.4%). The study found that participating mothers feared wizards and witches would terminate their pregnancy if they would be seen going for FANC visits; this resulted in a marginally significant number of participating women making less FANC visits. Apparently, witchcraft‐related myths are still prevalent in some parts of sub‐Saharan Africa. Mathole, Lindmark, Majoko, and Ahlberg (2004) also reported low utilization of FANC due to witchcraft‐related fears. This also highlights the need to intensify education to dispel myths and beliefs that impede progress on utilization of FANC.

The study elucidated that seeking permission from the husband or household head to attend antenatal care is significantly associated with low utilization of FANC (6.6%). Other studies showed that participating mothers who were waiting to seek permission made significantly fewer than the required number of visits for FANC (UNICEF 2008; Aarnio, Olsson, Chimbiri, & Kulmala, 2009). In this study, husbands (79%) mostly gave their wife's permission to start utilizing FANC. Male dominance in decision‐making on women's reproductive health requires more attention to minimize negative impact whilst maximizing desirable outcome. Thus, instead of women seeking permission they should ask for husband's involvement in antenatal care services. This idea is equally supported by Theuring, Nchimbi, Jordan‐Harder, and Harms (2009) who argues that pregnant women who first sought permission from husbands before utilizing FANC services are likely to make fewer than the required number of visits.

Postnatal mothers (85%) expressed concern that their husbands did not take an active role in FANC services. It was noted that although the husbands did not get involved in reproductive health activities, respondents stated plausible benefit if the husbands took an active role. Byamugisha et al. (2011) reported that attracting male partners in focused antenatal services (FANC) is very difficult. Their findings further revealed that male involvement in antenatal care services plays a major role as they make most decisions for their wives. Simkhada, Porter, and Van Teijlingen (2010) contended that mother‐in‐laws and mothers alone negatively influence the utilization of FANC services. However, Paredes, Hidalgo, Chedraui, Palma, & Eugenio, 2005 that low utilization of FANC services may not only be influenced by individual mother's characteristics, but also other social neighbourhood such as availability of services within reachable distances, inadequate media exposure and inadequate transport options due to lack of birth preparedness plans. Lee, Yin, and Yu (2009) support this notion by arguing that spouses and mother‐in‐laws persuade pregnant women to fulfil household duties instead of visiting antenatal care services.

The present study results are in accordance with the Health Belief Model. From the model, demographic and socio‐cultural factors associated with low utilization can be shown to be both modifying factors and perceived barriers that may affect health‐seeking behaviour of pregnant women. Parity and age are shown to be modifying factors of FANC utilization. Moreover, distance to the nearest health facility, witchcraft‐associated fear and seeking permission to go to the clinic is among perceived barriers to the utilization of FANC services by pregnant women in the Volta Region. Participating women had varied sources of information on FANC. The various sources of knowledge on FANC include midwife (51.4%), relatives (18.9%) and mass media (18.0%). Those who watched television and listened to the radio were more likely to use FANC services (Pallikadavath et al., 2004; Sharma, 2004). A statistically significant proportion of postnatal women (47.1%) indicated that they know nothing about the existence and importance of FANC which can negatively influence the utilization of FANC. As women who are expected to have completed the FANC services and given birth still have very little knowledge of the service, healthcare authorities will need to heighten campaign and tailor health information especially for the target groups. Knowledge about FANC would bring positive results in terms of utilization of the FANC services (Nisar & White, 2003).

A small proportion (52.9%) of postnatal women stated that they use FANC services as recommended by the Ghana Health Service and WHO. Focused antenatal care (FANC) provides a package of services that contributes to the health and well‐being of a woman throughout her pregnancy, childbirth and the postnatal period (Mamba et al., 2017). The results of this study also demonstrate the fact that respondents have adequate knowledge on the benefits of utilizing FANC services. One of the most prominent benefits cited by participating mothers was that FANC assists in creating a good rapport between health workers and service users. Rapport building amongst women using antenatal care services is a prerequisite for continuation of service utilization (Hollander, 1997). Adequate knowledge of FANC services would contribute to a reduction in maternal mortality rate (WHO, 2010). Postnatal mothers (64.9%) were aware that individualized health education amongst the service users assists in transferring of knowledge from service providers to pregnant women. Most participating mothers had indicated that four visits should be made when there is a problem; however, most indicated that more than four visits should be made when there is a general health‐ or pregnancy‐related problem. These results demonstrate that the general knowledge among participating mothers on FANC is quite high; nevertheless, knowledge is not translated into utilization as captured; thus. only a small proportion of participating mothers indicated appropriately utilizes FANC.

The study shows that most postnatal women in the Ho Teaching Hospital have less knowledge of the FANC. The major sources of FANC information for pregnant women in Volta Region are the midwives, relatives and radio. Thus, this study demonstrates that knowledge of FANC in general and its benefits are quite low among postnatal women in Ho Teaching Hospital. Parity and age are the only demographic factors associated with low utilization of FANC in pregnant women in Ho Teaching Hospital. Long distance to health facilities, fear of witchcraft as well as individual choice of not wanting to make many visits also contributed to low utilization of FANC among pregnant women. The general belief in this particular evil spirit has the potential and preventing early disclosure of pregnancy and may limit the chance of seeking early focused antenatal care service. Ethnographic studies from Mozambique and southern Tanzania illustrated, for example, that women at an early stage of pregnancy delayed ANC initiation purposely to protect the unborn from witchcraft and sorcery attacks of jealous neighbours and kin (Chapman, 2003; Gross et al., 2012; Haws et al., 2010). Seeking permission from the husband or the household head to utilize FANC significantly contributed to low utilization of FANC by pregnant women in Ho Teaching Hospital.

This study was mainly a descriptive cross‐sectional study that asked postnatal women to recall their experiences of focused antenatal services they sort for care when they were pregnant. In researchers that involve recall by participants, there is always a general risk of recall bias and the most appropriate method to avert this limitation is to have an observation of pregnant women as they sort focused antenatal care. Nonetheless, this study is useful as it was able to document the postnatal women's perspective of the care rendered to them during pregnancy in the study area. Due to the cross‐sectional nature of the study, it is difficult to establish cause–effect relationship between dependent and independent variables as this study mainly used administered questionnaire. Also, pregnant women were recruited when they sort postnatal care services, denoting that this cadre of women might have higher health awareness and therefore might have a relative positive view of focused antenatal care services than those who do not attend postnatal services. Future study is aimed at identifying the study population at home and work communities than those in a health facility.

## 5 CONCLUSIONS

The results showed that some postnatal women have no awareness of FANC even though FANC would assist the health worker to distribute information, education and communication materials. It is therefore imperative to increase public education using mass media channels to ensure hundred participation in FANC services. Married postnatal women and those cohabiting used FANC more compared with the single, divorced and widowed. Respondents with secondary, tertiary or no educational background used FANC more compared with those with primary and junior high educational background. On the part of the participants’ occupation, traders, students and civil servants patronized FANC more compared with those involved in business, farming and other occupations. There is therefore the need to encourage the provision of FANC services at homes and workplaces as most of the people who did not receive FANC services appear to be working in the informal sector of the economy. The primary healthcare intervention already in place in Ghana can be leveraged to ensure that these services are provided through community health nurses and community midwives. Long distance to the health facility, seeking permission to use FANC was significantly associated with low utilization of FANC.

Information, education and communication strategies aimed at promoting health‐seeking behaviours should be enhanced both at health facilities and community levels by the Ministry of Health and the Ghana Health Service. Some of the issues to be intensified should be dispelling myths associated with pregnancy, informing communities that any pregnant woman including teenagers who are pregnant are at risk and requires medical attention during the entire pregnancy period. Communities should further be informed that regardless of the age and parity all pregnant women must be supported to utilize FANC services. The message can be disseminated through already existing structures such as health awareness campaigns, open forum, drama and songs. Government, communities and other development partners should increase health infrastructure so that distance to the health facility can be reduced. This will allow pregnant women to fully benefit from FANC services. Communities should be assisted to come up with strategies which will promote utilization of FANC services, for instance, agreeing to reward families, adhering to maternal and child health established policies. There should be multi‐sectoral collaboration to the promotion of maternal and child health.

## CONFLICT OF INTEREST

The author declare that they have no competing interest.

## AUTHOR CONTRIBUTIONS

All the authors participated in conception, design, data collection and drafting of this manuscript. All authors approved the final manuscript for publication.

## ETHICAL APPROVAL

Ethical clearance was obtained from the Institute of Health Research, University of Health and Allied Sciences, to conduct this study (UHAS‐REC A.1 (19) 17‐18).
